# Editorial Change

**Published:** 1874-03

**Authors:** 


### Editorial.

### Editorial Change.

We have the pleasure of announcing the addition to our Editorial Staff of Dr. Edward N. Brush, who has been for the last two years Chief Assistant and upon whom has devolved most of the labor of editing the Journal.

The Senior Editor, who has had sole charge for the last thirteen years, is now obliged from increase of professional duties to accept an honorary and advisory position, not however in the least abating from his interest in an enterprise which has been his constant care, his untiring labor and his greatest pride for a long series of years.

The Buffalo Medical and Surgical Journal has grown old and strong under the fostering care of the profession. It has impartially recorded medical observation and progress; endeavored to promote harmony and concert of effort in the profession, spoken kindly of all, and looked charitably where it was impossible to approve, in a word, has aimed to advance the interests and promote the well-being of the entire medical profession. In return it has been sustained by the best men in its ranks and its practical value augmented by contributions of the greatest merit and value. The success of our enterprise of which we boast then, is not of editorial labor, but the product of professional thought and effort in others; and we take this opportunity to acknowledge indebtedness to our friends and to thank them for favors which we have no other means to repay. The senior editor while resigning the positive interests of the JOURNAL to one who can devote to it more time and thought. will ever cherish an unabated interest in everything that can promote its value and the general prosperity of the Medical Profession.

---

In assuming the duties of Assistant Editor of a Journal of the reputation in the profession which the BUFFALO MEDICAL AND SURGICAL JOURNAL enjoys, we do so with a full appreciation of the responsibilities and labors which that position entails upon its holder. Having this knowledge in view we make no promises for the future, for we think none are required. The earnest, observing, hard working and practical men in the profession have thus far lent their aid and support to the JOURNAL, and we are sure that they are not now ready to retire from the field. The Senior Editor in his Introductory thirteen years ago, said, that he looked to the members of the profession for the success of the JOURNAL, and it depends now none the less upon them· He has taken the opportunity to thank them for support and sympathy in the past; it is to be earnestly hoped that they will continue the same in the future.

There are cases of much interest occurring constantly in Buffalo and vicinity which should be reported in the JOURNAL. It is not to be understood that only rare and unique ones are desired; it is rather those of every day practice, such as are to be met by every physician, and upon which information may be thrown if well reported.

Awaiting then the continued and hearty co-operation of the profession, we take our Editorial Chair, stimulated by the past history of the JOURNAL, anxiously, yet hopefully awaiting its future record. B.

